# Dysregulation of ubiquitination modification in renal cell carcinoma

**DOI:** 10.3389/fgene.2024.1453191

**Published:** 2024-12-19

**Authors:** Hongjie You, Hui Zhang, Xiaofeng Jin, Zejun Yan

**Affiliations:** Department of Urology, The First Affiliated Hospital of Ningbo University, Ningbo University, Ningbo, Zhejiang, China

**Keywords:** E3 ubiquitin ligases, renal cell carcinoma (RCC), ubiquitination, PROTAC, immunotherapy, deubiquitinase

## Abstract

Renal cell carcinoma (RCC) is a malignant tumor of the renal tubular epithelial cells with a relatively high incidence rate worldwide. A large number of studies have indicated that dysregulation of the ubiquitination, including ubiquitination and dysregulation, is associated with the occurrence and development of RCC. This review focuses on several abnormal signaling pathways caused by E3 ligases and deubiquitinases. Additionally, we discuss research progress in RCC treatment by targeting key enzymes related to ubiquitination modifications.

## Introduction

Renal cell carcinoma (RCC) is one of the top ten most common cancers in the world, accounting for approximately 3% of all adult cancers, and its incidence is rapidly increasing (Padala et al., 2020), and exploration of its underlying mechanisms is still ongoing.

The balance between protein synthesis and degradation plays a crucial role in various cellular processes including DNA repair, cell proliferation, and apoptosis (Park et al., 2020). Protein degradation is primarily controlled by the ubiquitination modifications, which includes ubiquitination and deubiquitination. These processes are primarily by ubiquitin (Ub), ubiquitin-activating E1 enzymes, ubiquitin-conjugating E2 enzymes, E3 ubiquitin ligases, 26S proteasome, and deubiquitylase (Zhao et al., 2022). E1, E2, and E3 function in a coordinated manner to transfer Ub molecules to substrate proteins and tag them. Subsequently, the proteasome hydrolyzes the tagged proteins (Akutsu et al., 2016; Schofield and Ratcliffe, 2004). Deubiquitination refers to the process of reversing ubiquitination, where deubiquitylase remove Ub molecules from substrate proteins, thereby preventing protein degradation. The dynamic conversion between ubiquitination and deubiquitination is closely linked to various cellular functions, and its dysregulation can lead to a range of diseases Dysregulation of this process has significant implications in the occurrence and development of RCC.

Since numerous studies indicate that dysregulation of ubiquitination modifications is closely associated with the occurrence and progression of human cancers. Relevant studies have reported that dysregulation of ubiquitination modifications of key proteins in the HIF, ferroptosis, PI3K/AKT/mTOR, and p53 signaling pathways contributes to the development of RCC. E3 ubiquitin ligases and deubiquitylase play critical roles in ubiquitination modifications, and numerous studies have identified agonists or antagonists of E3 ubiquitin ligases and deubiquitylase as well as two novel molecular technologies developed based on ubiquitination modifications, namely Proteolysis Targeting Chimeras (PROTACs) and Deubiquitinase-targeting.

Chimeras (DUBTACs), which aim to regulate protein stability by targeting specific cellular factors. Therefore, targeting components of the ubiquitination modification pathway represents a promising strategy for RCC treatment. This review introduces the functional roles of RCC-associated E3 ubiquitin ligases and deubiquitylase, summarizes molecules targeting these enzymes, and discusses their potential therapeutic applications in RCC patients, particularly in combination with immunotherapy.

## The regulation of hypoxia-inducible factor (HIF) signaling pathway via ubiquitination modification in RCC

HIF is an oxygen-sensitive basic helix-loop helix transcription factor specifically responsible for regulating biological processes that facilitate both oxygen delivery and cellular adaptation to oxygen deprivation.

HIF has two subunits, namely, an oxygen-sensitive α-subunit, HIF-α, and a constitutively expressed β-subunit, HIF-β, consisting of the HIF complex. When cells experience hypoxia, stabilized HIF-α dimerizes with HIF-β in the nucleus to form transcriptionally activated HIFs that regulate a series of cellular processes, including energy metabolism, angiogenesis, erythropoiesis, cell proliferation apoptosis and so on (Schofield and Ratcliffe, 2004). There are three types of HIFs: HIF-1, HIF-2, and HIF-3 (the study of HIF-3 is unclear and is not described here). Normally, HIF-1α is expressed in most cell types in fully developed kidneys, whereas HIF-2α is mainly found in renal interstitial fibroblast-like cells and endothelial cells, both of which maintain normal renal physiological function (Wiesener et al., 2003). While, both HIF-1α and HIF-2α have been identified as renal cell carcinoma (RCC)-related factors, involving in the development of RCC.

In most cases, HIF-1α is an RCC tumor suppressor and HIF-2α is a carcinogenic factor in RCC. It is speculated that HIF-1α leads to tumor regression because HIF-1α is often lost or rearranged in RCC cell lines. In addition, downregulation of HIF-1α in RCC cells promotes tumor growth, and supplementing HIF-1α deficient cell lines with HIF-1α leads to tumor regression (Shen et al., 2011).

Notably, the HIF signal is strictly regulated by the ubiquitination modifications, and disturbance of the ubiquitination modification leads to disorders of the HIF signal, thus contributing to the occurrence and progression of RCC. In this section, we will summarize and discuss the functions of several E3 ubiquitin ligases, including Von Hippel–Lindau (VHL), hypoxia-associated factor (HAF), as well as deubiquitylases, such as BRCA1-associated protein 1(BAP1), and ubiquitin-specific peptidase 37 (USP37) in the HIF signaling pathway the mechanisms by which their dysregulation leads to RCC.

## The role of E3 ubiquitin ligases in the HIF signaling pathway in RCC

### VHL

VHL is a tumor suppressor, acting as a substrate adaptor, consisting of the VHL E3 ubiquitin ligase complex together with Cullin-2 (Cul-2), Elongins B/C (ELB/ELC), and RING-box (Kaelin, 2002). VHL comprises an α-domain and a β-domain. The α-domain has been shown to directly bind elongin C, which allows for the formation of the VHL–elongin B–elongin C complex, in which the β-domain is responsible for recognition and the substrates (Stebbins et al., 1999). The VHL E3 ubiquitin ligase complex plays an important role in oxygen delivery to regulate biological processes, such as cell proliferation and apoptosis. Notably, mutations and deletions of *VHL* gene may contribute to the accumulation of HIF-1α and HIF-2α.

Notably, HIF-1α and HIF-2α both increased in expression when VHL was inactivated initially. With time, HIF-2α was predominant and suppressed HIF-1α at the protein level in RCC. Thus, HIF-2α eventually predominates over HIF-1α in accelerating tumor progression in RCC (Raval et al., 2005).

**FIGURE 1:**
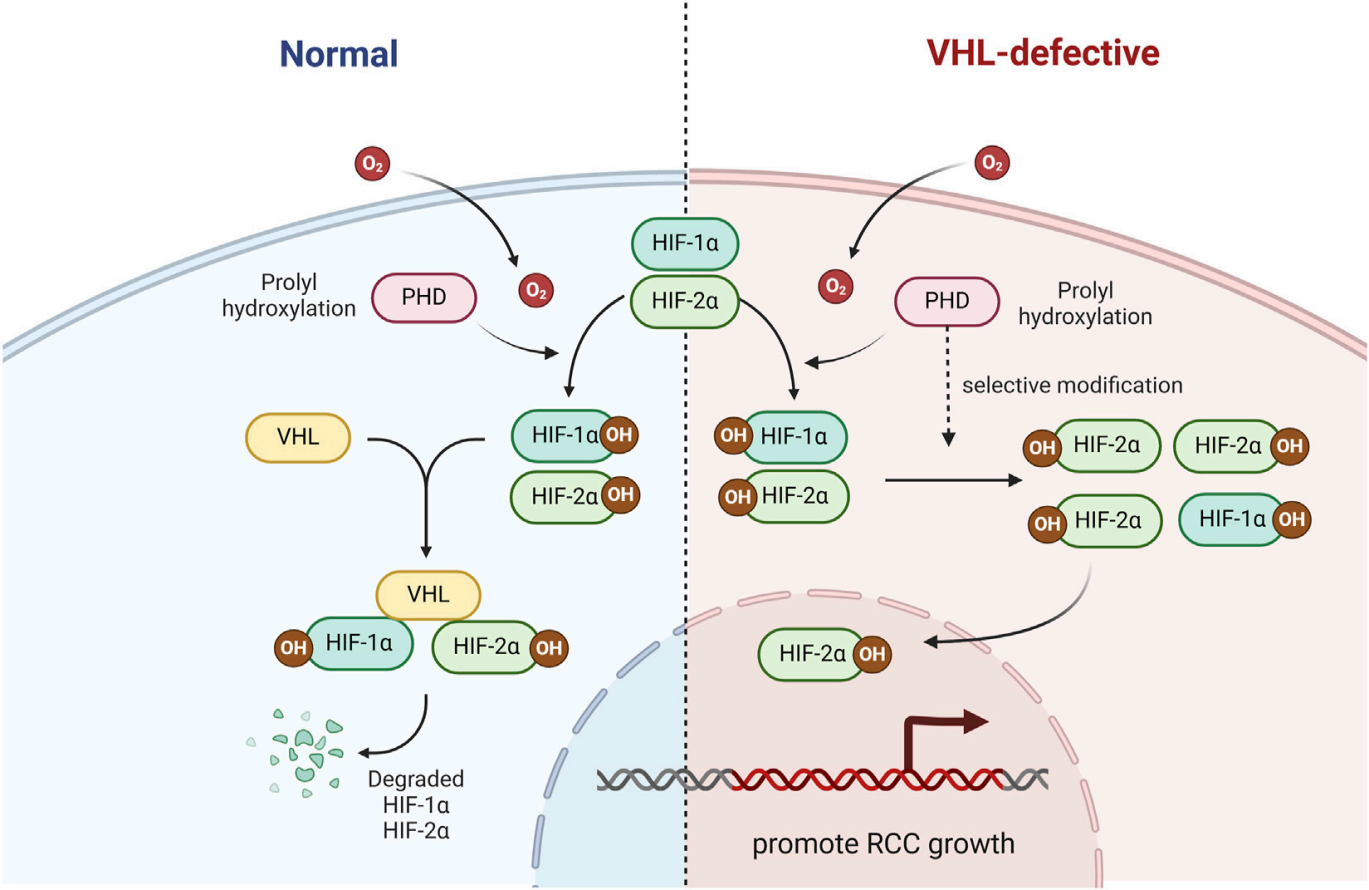
The role of VHL in the HIF signaling pathway. VHL ubiquitinate and degrade HIF-1α and HIF-2α.

At normal oxygen levels, HIF-α can undergo hydroxylation by active proline hydroxylases (PHD-1, PHD-2, and PHD-3) at specific proline residues. This hydroxylation modification allows the HIF-α subunit to be recognized and ubiquitinated by VHL (Schofield and Ratcliffe, 2005). Under hypoxic circumstances, the hydroxylation of HIF-1a is restricted by the disposal of oxygen molecules and HIF-1a is secured and assembles. HIF-1a can then dichotomize with HIF-b and prompt the transcription of hypoxia-survival genes (Ioannidou et al., 2021). Moreover, it leads to differences in the expression of HIF-1α and HIF-2α in RCC that PHD is selective for modification of HIF-1α and HIF-2α (Koh et al., 2008), which may explain why a large number of *VHL*-defective ccRCC cell lines do not express HIF-1α, but express HIF-2α (Figure 1).

In an additional *VHL*-knockout mouse model, we found that the risk of renal cyst formation, which is a precursor to clear cell renal cell carcinoma (ccRCC), depends on HIF-2α rather than HIF-1α, emphasizing the importance of HIF-2α as a cancerigenic factor (Rankin et al., 2006). In the meantime, a large number of VHL-defective ccRCC cell lines do not express HIF-1α, but express HIF-2α (Maxwell et al., 1999).

Furthermore, the epidermal growth factor receptor (EGFR) is common in human ccRCC and can also be involved in ccRCC progression. HIF-2α prolongs EGFR activity by inhibiting endocytosis and subsequent lysosomal degradation in *VHL*-deficient 786-O ccRCC cell lines (Zhou and Yang, 2011). Moreover, EGFR silencing efficiently blocked HIF-2α mediated tumorigenesis in *VHL*-knockout RCC cell lines (Smith et al., 2005). Cyclin D1 is a cell cycle regulator that plays an important role in RCC development, and is a promising target for HIF-2α in RCC cells. One related study showed that Cyclin D1 promotes tumor growth in *VHL-knockout* cancer cells in mice (Zhang et al., 2013). In summary, VHL plays an important role in the pathogenesis of RCC, and further research on VHL may benefit patients with RCC.

### HAF

HAF is a protein characterized by its modular structure, exhibiting DNA binding capability at its N-terminus and E3 ligase activity at its C-terminus (Koh and Powis, 2009). HAF, also known as SART1800 (squamous cell carcinoma antigen recognized by T cells), was originally identified as a nuclear protein expressed in proliferating cells (Shichijo et al., 1998). HAF was named after its capacity to attach to the promoter region of the erythropoietin (EPO) gene and trigger its transcription upon hypoxia (Gupta et al., 2000).

In addition, HAF is a HIF-1α specific E3 ligase that mediates its ubiquitination and degradation. After degrading HIF-1α, HAF binds to HIF-2α, increasing HIF-2α transactivating activity rather than leading to its degradation (Koh et al., 2011).

**FIGURE 2:**
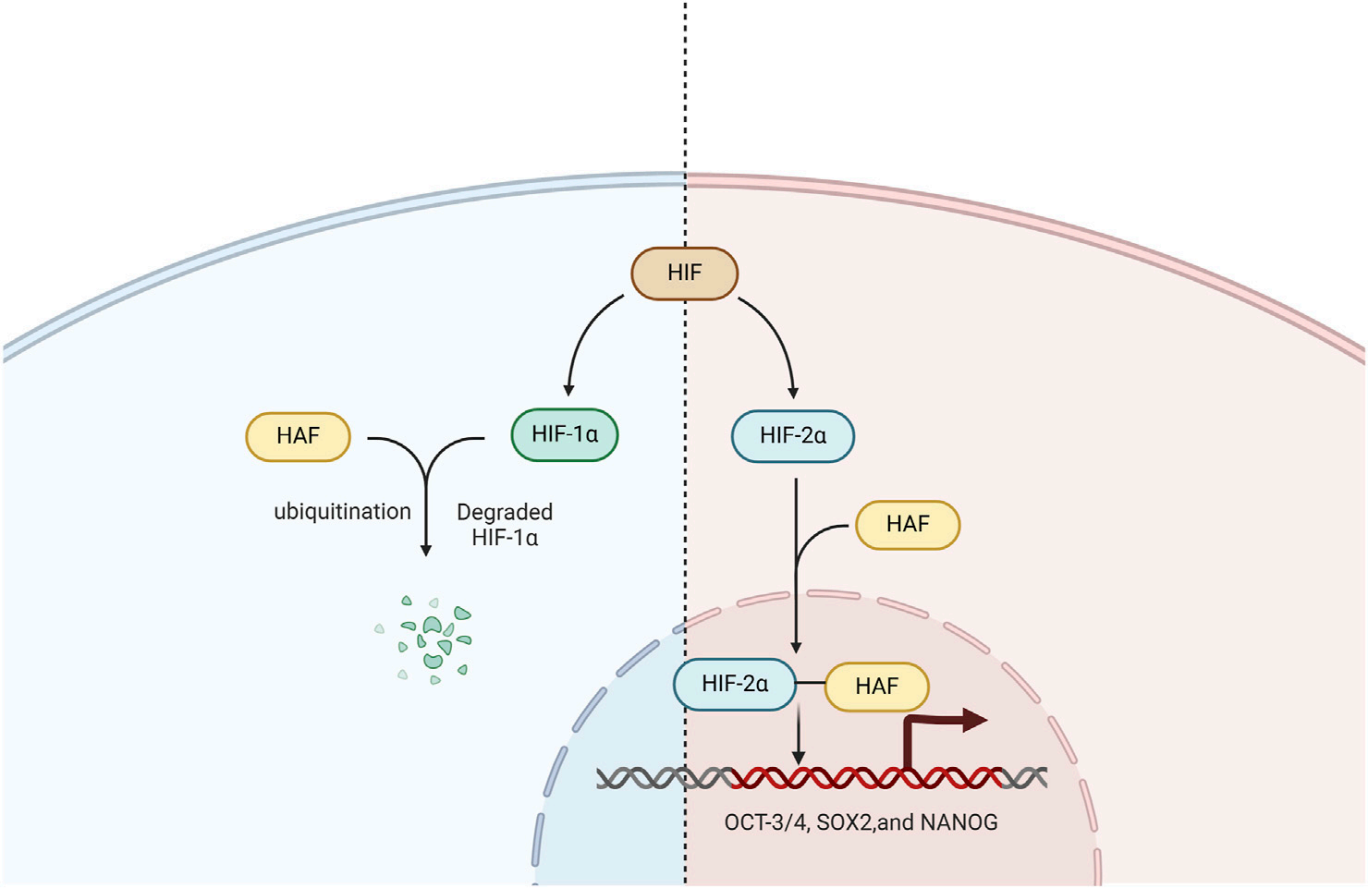
The role of HAF in the HIF signaling pathway. HAF mediates HIF-1α polyubiquitination and degradation, binds and activates HIF-2α, and promotes the transcription of HIF-2α target genes including OCT-3/4, SOX2, and NANOG.

In patients with ccRCC, the presence of HAF can significantly enhance tumor progression through ubiquitination and degradation of HIF-1α. Additionally, HAF has the ability to bind to and activate HIF-2α, driving the maximal expression of HIF-2α target genes (particularly of the HIF-2α dependent pluripotency genes *OCT-3/4*, *SOX2*,and *NANOG*), which can promote the enrichment of the cancer stem cell population, resulting in more aggressive tumors *in vivo* (Koh et al., 2015). Hence, targeting the HIF axis is a promising therapeutic strategy for ccRCC treatment (Figure 2).

## The role of deubiquitylase in HIF signaling pathway in RCC

### BAP1

BAP1 is a deubiquitylase that possesses a structure comprising an N-terminal ubiquitin carboxy-terminal hydrolase (UCH) domain and a host cell factor 1 binding motif (HBM) (Okino et al., 2015). BAP1 plays crucial roles in diverse cellular functions including histone modification, transcriptional regulation, chromosome stability, proliferation, DNA damage response, cell cycle progression, ER stress, metabolism, apoptosis, ferroptosis, cellular differentiation, and immune regulation (Langbein et al., 2022). In ccRCC, BAP1 is a tumor suppressor that plays a role in the suppression of cell proliferation, with mutations in approximately 15% of ccRCC cases. As a deubiquitylase, BAP1 serves as a critical regulator of the HIF signaling pathway, exerting a significant influence on ccRCC progression.

BAP1 is a key positive regulator of HIF-1α in hypoxia and binds to the N-terminal region of HIF-1α, where HIF-1α recognizes DNA and dimerizes with HIF-1β to form the heterodimeric transactivating complex HIF. Moreover, they found that BAP1 functions as a deubiquitylase, forming a complex that binds to and prevents its degradation of HIF-1α (Bononi et al., 2023). Therefore, testing the function of ccRCC-associated BAP1 mutations may help to explain the underlying mechanism by which dysregulation of BAP1- HIF-1α leads to the progression of ccRCC.

### USP37

**FIGURE 3:**
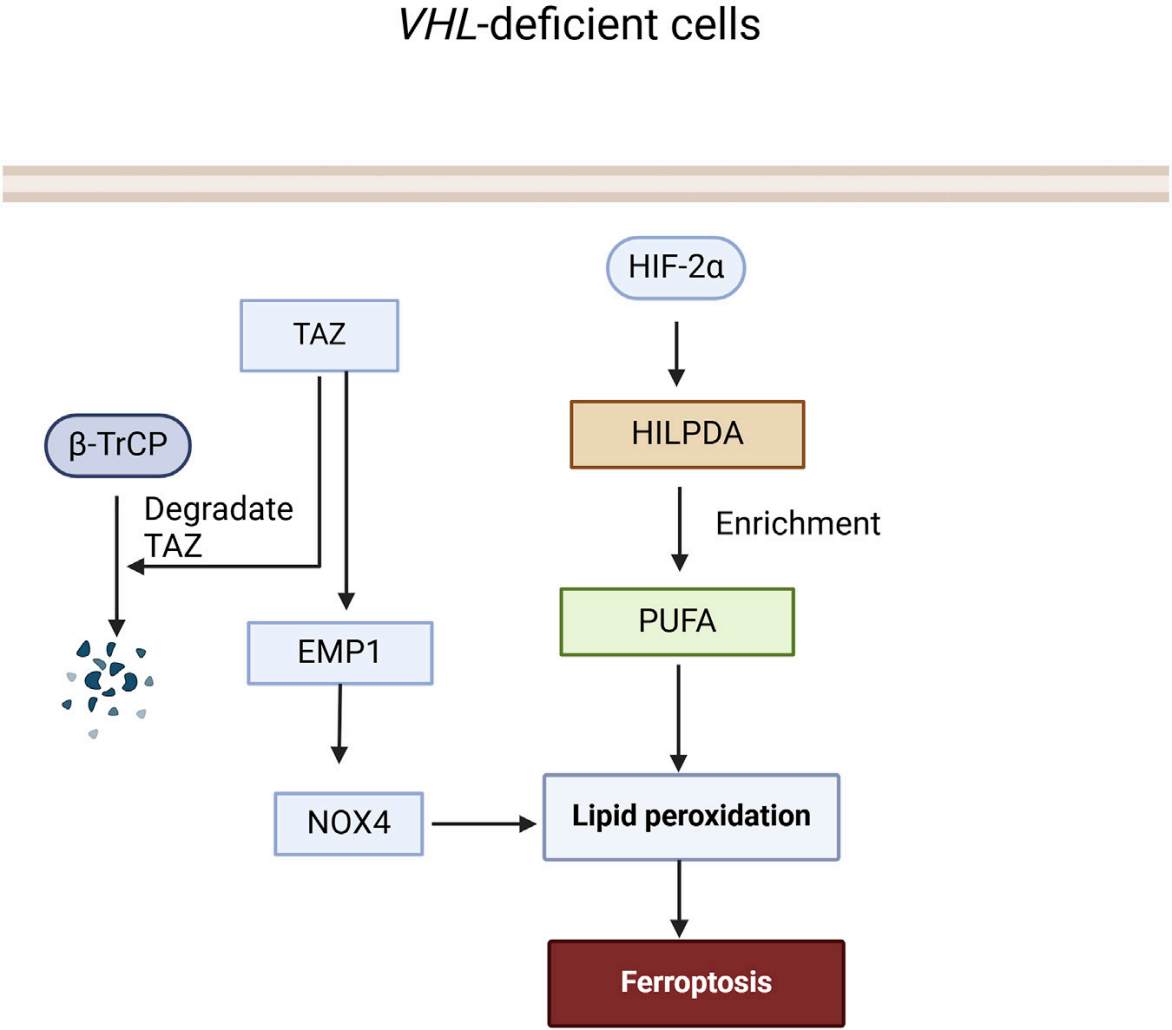
The role of BAP1 and USP37 in the HIF signaling pathway. BAP1 deubiquitinates and binds to stabilize HIF-1α, facilitating its association with HIF-1β to promote transcriptional activity in cells. USP37 deubiquitinates and binds to stabilize HIF-2α, thereby enhancing the transcription of its target genes.

USP37, a member of the ubiquitin-specific processing protease family, consists of 979 amino acids and contains three ubiquitin-interacting motifs located between the Cys box and His box within its primary sequence (Qin et al., 2018). USP37 is a deubiquitinase that binds HIF-2α and facilitates HIF-2α deubiquitination to maintain HIF-2α stability. One study tested multiple independent USP37 small interfering RNAs (siRNAs) in several ccRCC cell lines, including UMRC2, 786-O, and UMRC6 cells. USP37 depletion led to decreased HIF-2α protein levels in these cells, suggesting that USP37 is essential for the regulation of HIF-2α in ccRCC cells. Moreover, they also found that USP37 plays an essential role in maintaining kidney tumorigenesis in an orthotopic xenograft model using HIF-2α, and the depletion of USP37 results in reduced primary kidney tumorigenesis (Hong et al., 2020). Hence, inhibitors targeting USP37 may be a potential approach for treating patients with RCC (Figure 3).

## The regulation of ferroptosis via ubiquitination modification in RCC

Ferroptosis, different from other types of cell death, is a novel iron-dependent programmed cell death pattern characterized by iron metabolism disorders, accumulation of reactive oxygen species (ROS), and lipid peroxides. Ferroptosis can be triggered by an excessive amount of catalytic iron that can initiate lipid peroxidation, leading to the Fenton reaction (which can generate a large quantity of hydroxyl free radicals to attack cells and trigger ferroptosis) (Wang X. et al., 2021). The regulatory mechanisms of ferroptosis are complex; glutathione depletion and GPX4 activity decline drive the hyperperoxidation of lipids, leading to ferroptosis in biochemical terms (Ding et al., 2020). Generally, ferroptosis mechanisms are mediated by many regulatory factors (Nie et al., 2018), including changes in iron homeostasis, lipid peroxidation, and amino acid metabolism.

Emerging evidence has demonstrated the sensitivity of ccRCC to ferroptosis, suggesting that inducing ferroptosis could be a novel approach for treating patients with ccRCC (Chang et al., 2021). Moreover, several studies have demonstrated that ubiquitination is also involved in ferroptosis. Therefore, in this section, we will summarize and discuss E3 ligases such as VHL and β-transducin repeat-containing protein (β-TrCP), as well as other deubiquitinases in ferroptosis, and their dysregulation in relation to the development of RCC.

## The role of E3 ubiquitin ligases in ferroptosis in RCC

### VHL

Current research suggests that VHL can effectively inhibit ferroptosis by reversing the cell’s metabolism to an oxidative one and reducing lipid peroxide accumulation. Notably, the number, size, and intensity of lipid droplets in cells with VHL restoration were significantly reduced compared with *VHL*-deficient cells. Mechanistically, VHL promotes the ubiquitination and degradation of HIF-2α, thus increasing the polyunsaturated fatty acids (PUFA), which are suppressed by HIF-2α, a lipid more susceptible to peroxidation, by activating the expression of hypoxia-inducible and lipid droplet-related protein (HILPDA) (Miess et al., 2018), finally contributing to ferroptosis in *VHL*-deficient ccRCC.

### β-TrCP

β-TrCP, an F-box protein, consists of the SCF^β−TrCP^ E3 ubiquitin ligase complex, together with a scaffold protein CUL1, a RING protein RBX1, and an adaptor protein SKP1. β-TrCP, a substrate receptor, controls ubiquitination and proteasomal degradation. β-TrCP plays a vital role in regulating various physiological and pathological mechanisms, including signal transmission, cellular division, the cell cycle, programmed cell death, genetic stability, and tumorigenesis (Bi et al., 2021).

**FIGURE 4:**
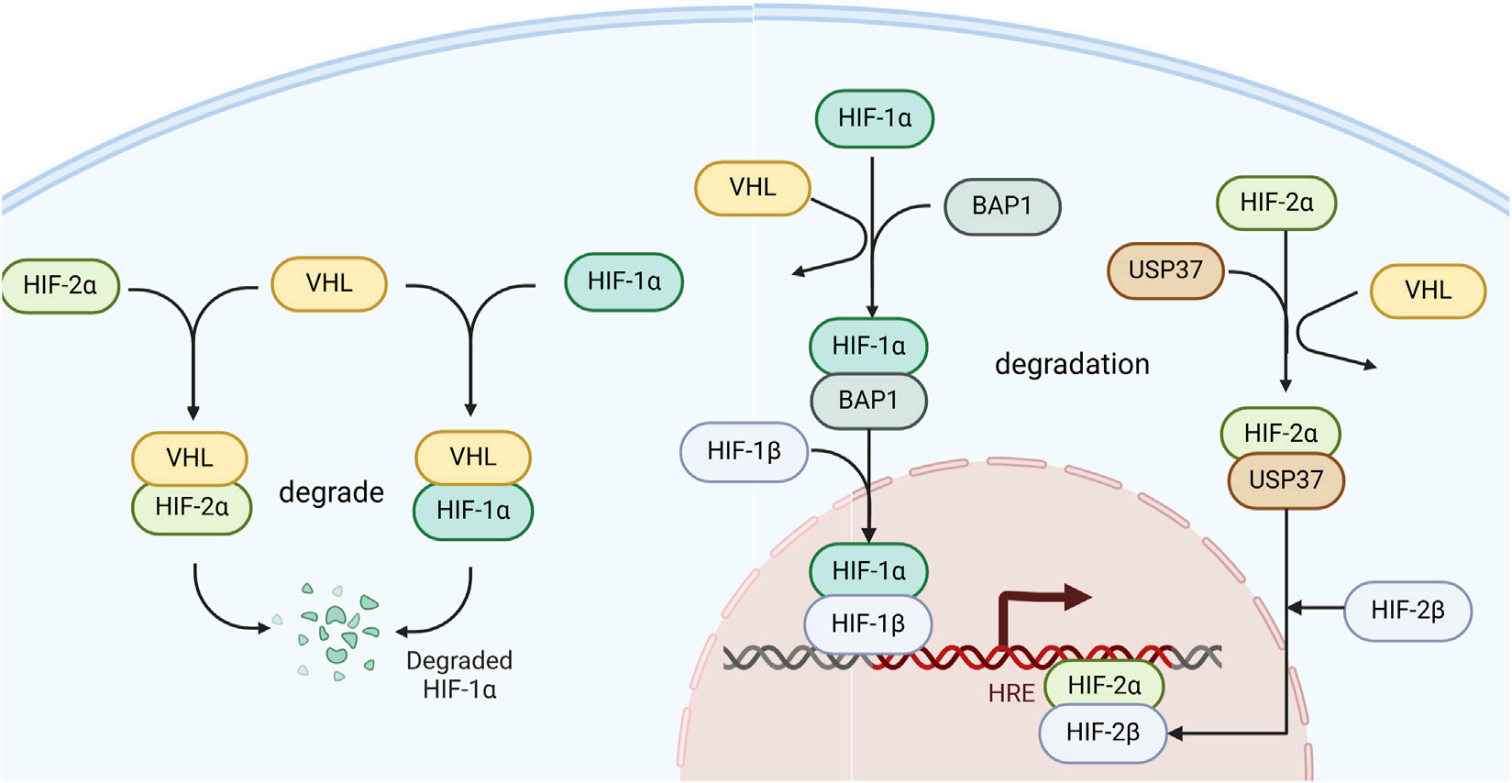
The role of VHL and β-TrCP in the Ferroptosis signaling pathway. In VHL-deficient cells, HIF-2α is not ubiquitinated and degraded by VHL, leading to an increase in HILPDA expression and accumulation of PUFA, which in turn promotes the accumulation of lipid peroxides and triggers ferroptosis. TAZ can induce lipid peroxide accumulation and trigger ferroptosis through the EMP1-NOX4 pathway, while β-TrCP can ubiquitinate and degrade TAZ to terminate this process.

In RCC, β-TrCP primarily influences the metabolism of iron ions and ferroptosis within renal cells through ubiquitination and degradation of the Transcriptional Coactivator with PDZ-binding Motif (TAZ). *TAZ* knockdown in RCC cells resulted in decreased expression of epithelial membrane protein 1 (EMP1), leading to cellular resistance to ferroptosis. Silencing EMP1 also conferred resistance to ferroptosis in RCC cells and decreased the expression level of nicotinamide adenine dinucleotide phosphate oxidase 4 (NOX4), which can influence lipid peroxidation and affect sensitivity to ferroptosis (Yang et al., 2019). In summary, β-TrCP regulates ferroptosis in RCC by ubiquitinating and degrading TAZ, which controls EMP1-NOX4 signaling (Figure 4).

## The role of deubiquitylase in ferroptosis in RCC

### BAP1

**FIGURE 5:**
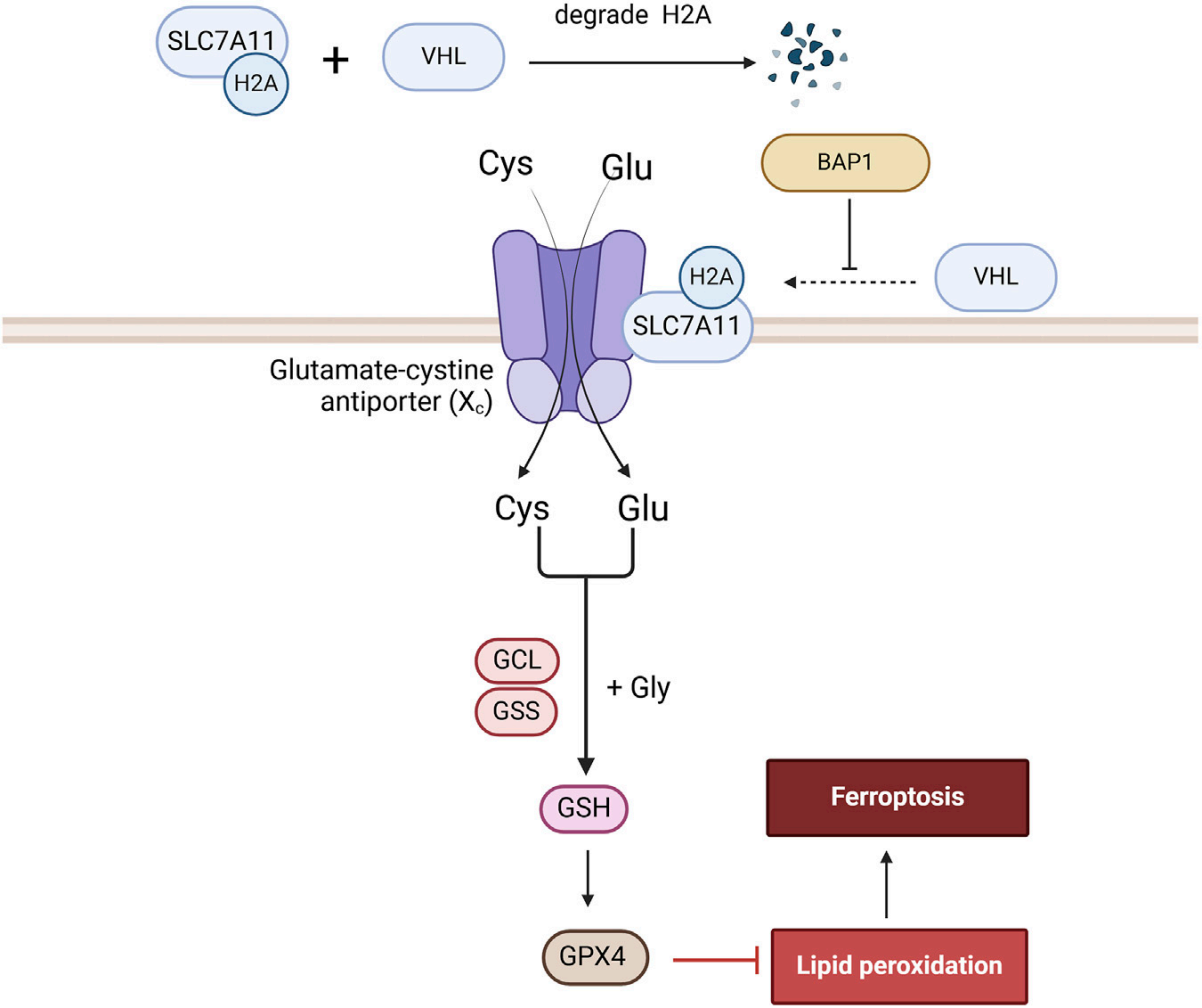
The role of BAP1 in the Ferroptosis signaling pathway. BAP1 promotes ferroptosis by ubiquitinating H2A on the SLC7A11 promoter, a component of the Xc-transporter, thereby reducing glutathione synthesis and GPX4 generation.

BAP1 represses solute carrier family 7 member 11 (SLC7A11) expression by reducing H2A ubiquitination of the SLC7A11 promoter, resulting in lipid peroxidation and ferroptosis (Zhang et al., 2019). SLC7A11 is a constituent of the Xc-transportermediated system that can import extracellular cysteine into cells to promote glutathione synthesis, thereby inhibiting ferroptosis (Wang X. et al., 2021). A study found that SLC7A11 not only significantly influenced RCC prognosis and the tumor immune microenvironment, but also facilitated RCC progression by inhibiting ferroptosis and enhancing metabolic reprogramming. Moreover, SLC7A11 enhances RCC proliferation, migration, and invasion by increasing GPX4 activity, thereby inhibiting ferroptosis (Xu et al., 2021) (Figure 5).

## The regulation of PI3K/AKT/mTOR signaling pathway via ubiquitination modification in RCC

The phosphatidylinositol 3-kinase (PI3K)/protein kinase B (AKT)/mammalian target of rapamycin (mTOR) signaling pathway is involved in regulating essential biological processes, such as cell growth, proliferation, apoptosis, and cellular metabolism, and plays an important role in cancer. Abnormal activation of the PI3K/AKT/mTOR signaling pathway leads to the occurrence and progression of human cancers.

Activated PI3K catalyzes the conversion of phosphatidylinositol 4,5-bisphosphate (PIP2) to phosphatidylinositol 3,4,5-triphosphate (PIP3). PIP3 activates AKT, causing it to translocate from the cytoplasm to the cell membrane where it becomes phosphorylated and activated (Miricescu et al., 2021). Activated AKT can phosphorylate mTOR, its downstream target. This activation makes mTOR a key regulator of cell metabolism. There are two distinct activated multiprotein complexes, mTORC1 and mTORC2 (Guertin and Sabatini, 2007). mTORC1 phosphorylates downstream targets to promote protein synthesis and cell growth. mTORC2 is involved in the phosphorylation and activation of AKT, further enhancing the activity of the AKT signaling pathway (Glaviano et al., 2023). Abnormal activation of the PI3K/AKT/mTOR pathway is frequently observed in RCC, which promotes the growth, proliferation, and metastasis of cancer cells. In this section, the key role of ubiquitination in the PI3K/AKT/mTOR signaling pathway and the mechanisms by which their dysregulation leads to RCC are comprehensively summarized.

## The role of E3 ubiquitin ligases of PI3K/AKT/mTOR in RCC

### VHL

VHL also inhibits the PI3K/AKT/mTOR signaling pathway. VHL can directly or indirectly affect RCC via the PI3K/AKT/mTOR signaling pathway. VHL can directly inhibit AKT activity, thereby affecting RCC progression. In *VHL*-knockout RCC cell lines, AKT is activated, promoting cell survival and tumor development (Guo et al., 2016). Furthermore, the regulatory associated protein of mTOR (RAPTOR) is a subunit of the mTORC1 complex, together with mTOR and mLST8. RAPTOR is a novel target of VHL E3 ubiquitin ligase, which can be ubiquitinated and degraded by VHL, thereby preventing the mTORC1 signaling pathway in ccRCC (Ganner et al., 2021). In contrast, VHL can influence the PI3K/AKT/mTOR axis through HIF-dependent mechanisms. In VHL-defective ccRCC cell lines, a large amount of HIF-2α enhancing mTORC1 activity by upregulating the expression of the SLC7A5 amino acid carrier under conditions of low amino acid availability (Elorza et al., 2012).

### Other E3 ubiquitin ligases

In addition to VHL, the E3 ubiquitin ligase ZNRF1 has been shown to participates in the ubiquitination and degradation of AKT in RCC cells. Moreover, a previous study showed that ZNRF1 interacts with Leucine Zipper Transcription Factor-like 1 (LZTFL1), which is a tumor suppressor that inhibits the G1 to S phase cell cycle transition by destabilizing AKT (Lu et al., 2023). F-box and WD-40 domain protein 7 (FBXW7) is an ubiquitin ligase E3 that can ubiquitinate and degrade mTOR. A previous study demonstrated that mTOR protein expression was reduced in RCC cells upon transfection with the FBXW7 expression vector (Okazaki et al., 2014).

## The role of deubiquitylase of PI3K/AKT/mTOR in RCC

### OTUD1 AND USP46

Deubiquitylase ovarian tumor deubiquitinase 1(OTUD1) and Ubiquitin-specific peptidase 46 (USP46) have been shown to decrease the phosphorylation of AKT at its N-terminus. The OTUD1 C-terminal deubiquitinase domain decreased Akt K63-linked polyubiquitination, thereby coordinating the inhibitory effect of the N-terminus. OTUD1 deubiquitinates and stabilizes PTEN, a well-known negative regulator of the PI3K/AKT/mTOR signaling pathway in ccRCC (Liu et al., 2022c). USP46 decreases the phosphorylation levels of AKT to inhibit cell proliferation and migration in RCC cells (Gui et al., 2019).

**FIGURE 6:**
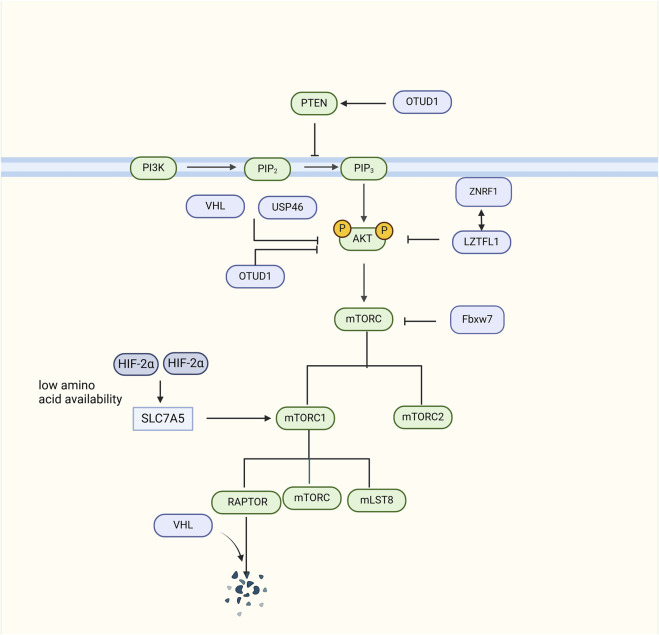
The role of Deubiquitylase in the PI3K/AKT/mTOR signaling pathway signaling pathway. VHL can ubiquitinate and degrade RAPTOR, an integral component of the mTORC1 complex, thereby inhibiting the AKT signaling pathway. VHL can also directly inhibit AKT activity to inhibit the AKT signaling pathway. In VHL-deficient cells, HIF-2α upregulates SLC7A5 amino acid carrier expression to enhance mTORC1 activity. ZNRF1 can polyubiquitinate and degrade AKT, and also interacts with LZTFL1 to inhibit AKT. Fbxw7 ubiquitinates and degrades mTOR. USP46 inhibits AKT levels. OTUD1 deubiquitinates and stabilizes PTEN, and regulates its inhibitory effect on AKT through deubiquitination.

Collectively, multiple E3 ubiquitin ligases and deubiquitylases have been shown to play critical roles in RCC development by participating in the ubiquitination of critical components of the PI3K/AKT/mTOR signaling pathway (Figure 6).

## The regulation of p53 signaling pathway via ubiquitination modification in RCC

p53 is a tumor suppressor protein consisting of an N-terminal activation domain, a proline-rich domain, a DNA-binding domain, an oligomerization domain, and a C-terminal domain. p53, also known as the guardian of the genome, can stabilize and activate various cellular stresses. It regulates multiple biological responses, such as cell cycle arrest, DNA repair, autophagy, ferroptosis, and the DNA repair response, inducing genes to restore cell homeostasis or initiate apoptosis (Amendolare et al., 2022). Functional inactivation of p53 protein has been identified in approximately 90% of human cancers, including RCC. In this section, the key roles of ubiquitination in the p53 signaling pathway and the mechanisms by which their dysregulation leads to RCC are comprehensively summarized.

## The role of E3 ubiquitin ligases of p53 in RCC

### MDM2

Murine double minute 2 (MDM2) is an amino acid regulator protein that consists of an N-terminal p53 binding domain, an acidic domain, a zinc-finger domain, and a C-terminal RING-finger domain that has E3 ligase activity (Koo et al., 2022). *MDM2* is a p53 target gene and the main negative regulator of p53 stability via the ubiquitination modifications. The N-terminal p53-binding domain of MDM2 interacts with the N-terminal domain of p53, allowing the C-terminal RING finger domain of MDM2 to function as an E3 ubiquitin ligase, inducing the ubiquitination and degradation of p53 (Inoue et al., 2016).

In normal cells, a dynamic equilibrium exists between p53 and MDM2 that maintains cellular homeostasis. However, in ccRCC cells, the expression of wild-type *p53* is abnormally low, primarily because of the activation of MDM2 by mTOR, leading to ubiquitination and degradation of wild-type p*53*, thereby promoting ccRCC (Roe and Youn, 2006). Additionally, in *VHL*-deficient RCC cells, siRNA-mediated downregulation of MDM2 results in decreased protein levels of HIF-1α and HIF-2α while increasing the expression of VEGF and plasminogen activator inhibitor-1 proteins (Carroll and Ashcroft, 2008). A related study showed that the combined use of MDM2 inhibitors and everolimus reduced the size of *in vivo* and *in vitro* ccRCC models (Dell'Atti et al., 2022). In summary, the ubiquitination function of MDM2 has a significant impact on RCC, and targeting MDM2 holds great promise for RCC treatment.

### Other E3 ubiquitin ligases

Tripartite motif-47 (TRIM47), a member of the TRIM protein family, has a RING domain that can play a role in E3 ligase activity. One related study showed that tumor growth significantly slowed in mice injected with *TRIM47*-knockout cells. Moreover, they demonstrated that TRIM47 could interact with p53 in RCC, so that elevated TRIM47 expression could enhance ubiquitination and degradation of p53, leading to the malignant biological behavior of RCC (Chen et al., 2021). RANBP2-type and C3HC4-type zinc finger-containing 1 (RBCK1) consist of an N-terminal ubiquitin-like domain, Npl4-type zinc finger domain, and catalytic C-terminal RBR domain. One related study demonstrated that RBCK1 promoted the polyubiquitination and degradation of p53 by directly interacting with it, and RBCK1 depletion affected proliferation, apoptosis, and cell cycle arrest in RCC cells, possibly due to RBCK1-mediated ubiquitination and degradation of p53 (Yu et al., 2019).

## The role of deubiquitylase of p53 in RCC

### USP10

USP10, a member of the ubiquitin-specific processing protease family, consists of an N-terminal ubiquitin-associated domain, a conserved ubiquitin-specific protease domain in the central region, and additional functional domains at the C-terminal end (Ye et al., 2022). USP10 interacts with p53 and deubiquitinates p53 to stabilize it. USP10 counteracts the degradation of p53 by MDM2, thereby maintaining the dynamic equilibrium of p53.

**FIGURE 7:**
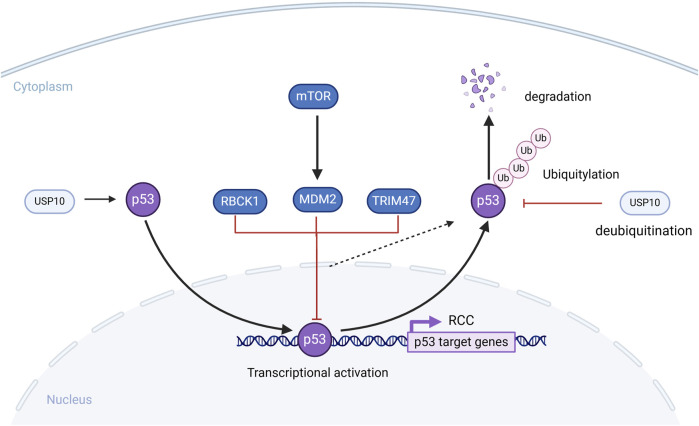
The role of Deubiquitylase in the p53 signaling pathway signaling pathway. MDM2, TRIM47, RBCK1 can ubiquitinate and degrade p53, which affects the development of RCC cells. USP10 deubiquitinates p53 and stabilizes it, counteracting the degradation of p53 by MDM2.

A related study showed that reconstituting USP10 in RCC cells with downregulated USP10 restores p53 expression and inhibits colony formation as well as cell proliferation in RCC cells. However, they also found that in RCC cells with wild-type p53, USP10 functions as a tumor suppressor, whereas in RCC cells with p53 mutations, USP10 has a tumor suppressive role in promoting cancer occurrence and progression of cell growth (Yuan et al., 2010) (Figure 7).

### Treatment

Currently, the main treatment for RCC is surgery; however, approximately 30% of patients experience cancer recurrence after surgery (Makhov et al., 2018). In the past few years, drug treatment for RCC has transitioned from cytokine therapy to small molecule kinase inhibitor-targeted therapy, and now to the era of immune checkpoint inhibitor therapy (Barata and Rini, 2017). The first-line treatment for RCC includes tyrosine kinase inhibitors (TKIs), immune checkpoint inhibitors (ICIs), or combinations of ICIs (Bedke et al., 2021). These drugs have indeed improved the survival rates of patients with RCC, but the issue of drug resistance after treatment still persists. Multi-targeted tyrosine kinase inhibitors can lead to various adverse events, among which hematologic and hepatic toxicities are particularly significant. However, there is currently no definitive evidence to support a relationship between the severity of adverse events and efficacy. Additionally, the TKI pazopanib may also induce depigmentation, diarrhea, nausea, anorexia, vomiting, and even posterior reversible encephalopathy syndrome (Savaliya et al., 2023). Therefore, there is an urgent need for further exploration of the mechanisms underlying RCC drug resistance, thus providing a promising treatment for RCC patients. Dysregulation of ubiquitination modifications is one of the key mechanisms in the development of RCC. Imbalances in E3 ubiquitin ligases and deubiquitylase can lead to the disruption of multiple intracellular signaling pathways, such as HIF, ferroptosis, PI3K/AKT/mTOR, and p53, ultimately resulting in uncontrolled tumor cell proliferation. Therefore, targeting E3 ubiquitin ligases and deubiquitylase to maintain the stability of ubiquitination modifications represents a feasible and promising therapeutic approach for RCC.

**FIGURE 8:**
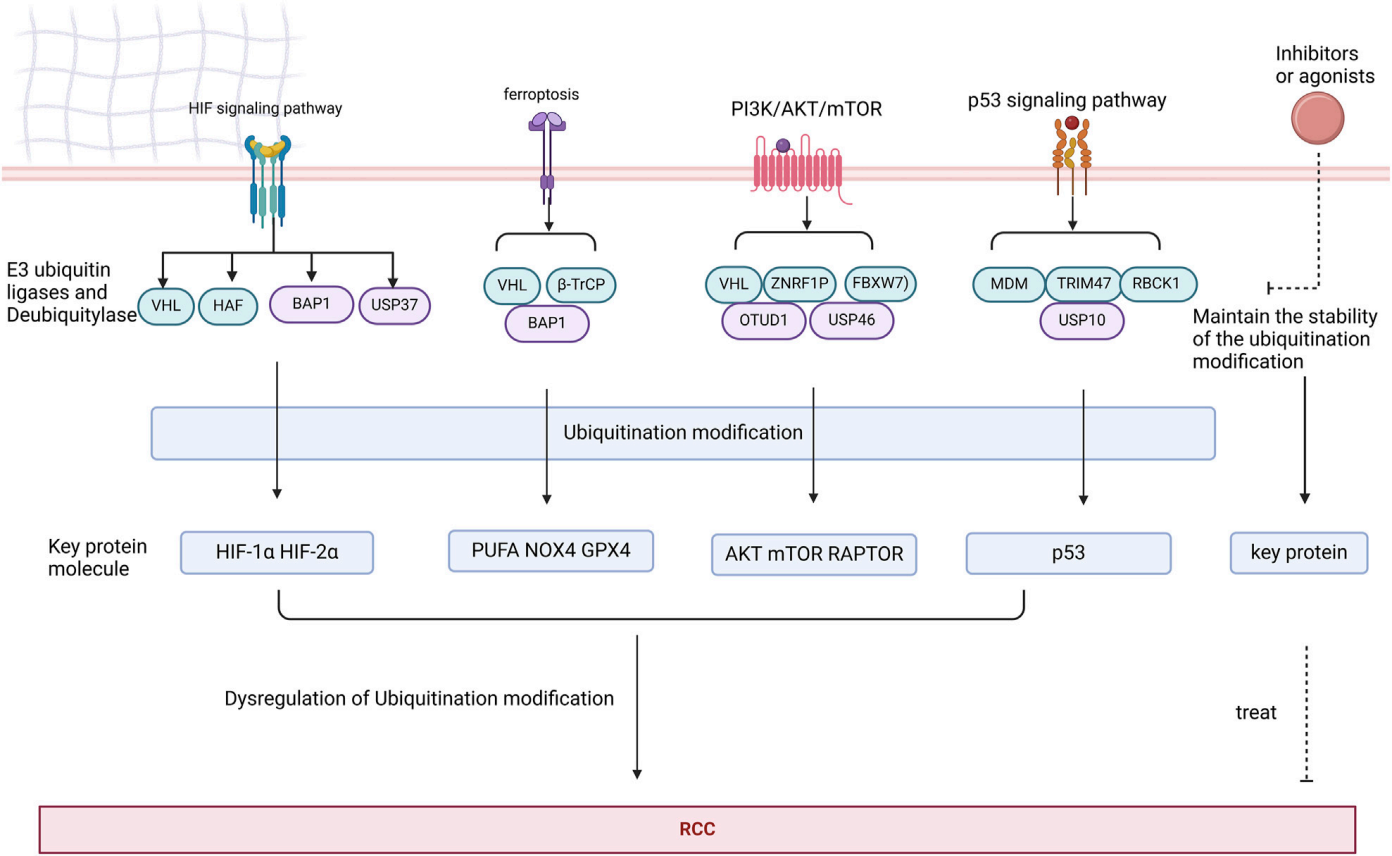
Key proteins in the HIF, ferroptosis, PI3K/AKT/mTOR, and p53 signaling pathways are subjected to ubiquitination modifications by deubiquitinases and E3 ubiquitin ligases. Dysregulation of these ubiquitination modifications may contribute to the development of RCC. Targeting agonists or inhibitors of deubiquitinases and E3 ubiquitin ligases represents a potential therapeutic strategy for RCC.

PROTAC, ubiquitination modification and DUBTAC have gradually become hotspots for overcoming these problems. Given that clinical trials and applications of Ubiquitination modification inhibitors are still lacking, this section focuses on Ubiquitination modification inhibitors targeting immunotherapy, PROTAC and DUBTACs (Figure 8).

## Ubiquitination modification and immunotherapy combination in RCC

Cancer cells can take advantage of signaling through the interaction of immune factors, such as cytotoxic T-lymphocyte-associated protein 4 (CTLA-4), which binds to the B7 ligand, and programmed cell death protein 1 (PD-1), which binds to programmed death ligand-1 (PD-L1) to promote tumor development while evading attacks from the immune system. The principle of immunotherapy is ICIs, which can prevent the signaling of immune system being suppressed, thereby stimulating it to destroy tumor cells (Jang et al., 2021).

RCC exhibits a significant and intricate infiltration of immune cells and widespread expression of PD-L1 (Deleuze et al., 2020), which has the potential to serve as a reliable biomarker for immunotherapies (Considine and Hurwitz, 2019). Immunotherapeutic agents targeting PD-1/ PD-L1, such as Nivolumab and Ipilimumab, have demonstrated superior long-term survival benefits compared with sunitinib, a TKIs, in untreated intermediate/low-risk advanced RCC patients (Boussios et al., 2023). However, ICIs have not yet been fully developed and have limitations. For example, owing to the complex tumor microenvironment, only a small subset of patients with RCC can benefit from these drugs (Sammarco et al., 2023). Additionally, there are other constraints, such as short-lived anti-tumor responses, drug resistance, and adverse reactions in the body. Therefore, researchers are constantly researching new ICIs drugs or employing combination therapies, such as combining ICIs with different ICIs or ICIs with TKIs, to avoid the limitations of immunotherapy.

In addition to combination therapies and new drugs, ubiquitination modification are closely associated with the immune pathway. Therefore, we have summarized the potential combination of ubiquitination modification inhibitors with ICIs, such as targeting Cbl-b and FBXW7 in RCC (Table 1).

**TABLE 1:** Reported inhibitors of ubiquitination modification in RCC.

Kind	Targets	Compound	Mechanism of action	Global status	Refere nce
E3 ubiquitin ligases	Cbl-b	NCT02166255	Interfering RNA reduces expression	Preclinical	Gavali et al. (2021)
		NCT03087591	Interfering RNA reduces expression	Preclinical	Gavali et al. (2021)
		NX-0255	Activating CD8^+^ T cells targeting specific antigens	Preclinical	August in et al. (2023)
		HOT-A	Inhibit phosphorylation of Cbl-b	Preclinical	Kuai et al. (2021)
	FBXW7	Genistein	Inhibit miR-223	Preclinical	Ma et al. (2013)
		Decitabine	Non-methylated promoter	Preclinical	DiNar do et al. (2018)
		ABT199 and BI2536	Phosphorylation of FBXW7	Preclinical	(Fan et al., 2022)
Deubiquityla se	USP7	P5091	Reduction of Foxp3 levels in CD4^+^ T cells	Preclinical	Fu et al. (2019)
		Almac4	weakening the interaction between PD-L1 and PD-1	Preclinical	Wang Z. et al. (2021)
	CNS5	Curcumin	Inhibition of CNS5 ubiquitin ligase activity	Preclinical	Uhle et al. (2003)
		Berberine	Specific binding to Glu-76 of CSN5	Preclinical	Liu et al. (2020b)
		CSN5i-3	Inhibition of the SRC family signaling.	Preclinical	Mazzu et al. (2022)

## The role of E3 ubiquitin ligases in RCC therapy

### Cbl-b

Cbl-b, a RING finger E3 enzyme, is capable of facilitating the transfer of ubiquitin from E2 enzymes to target proteins (Lu et al., 2022). In immune activation, CBL-B, a crucial checkpoint, acts as a non-redundant negative modulator and plays a key role in T cell activation. Its function involves increasing the threshold for T cell activation through ubiquitination and disrupting critical T cell signaling components associated with the T cell receptor pathway and immune synapse rearrangements (Weissman et al., 2011). CBL-B can mediate the ubiquitination and degradation of PD-1, further inhibiting tumor development and immune infiltration. Current research on the relationship between CBL-B and RCC is relatively limited. A related study showed that the collaboration between c-Cbl and VHL in *VHL*-knockout RCC cell lines resulted in the downregulation of activated EGFR when c-Cbl was downregulated (Paolino and Penninger, 2010). Indeed, during T-cell activation, the PD-1/PD-L1 pathway promotes the accumulation of CBL-B. However, inhibitors of CBL-b antagonize immune suppression in cancer cells. Thus, because of its multiple checkpoint inhibitory roles and minimal autoimmune toxicities, CBL-B is a strong candidate for targeted cancer immunotherapy.

Cbl-b is a potential target for RCC therapy, but current Cbl-b inhibitors are mainly in the preclinical stage of development because of their unfavorable chemical structures, competitive active sites, and elusive allosteric pockets. Scientists have effectively reduced CBL-B expression in T cells through RNA interference, exhibiting promising outcomes in various adoptive T-cell transfer tumor models. Gien that the good effect among cancer patients, these approach, The adoptive cell transfer therapy combining DC vaccination with *ex vivo* Cbl-b silencing has successfully slowed the growth of tumor cells, and a Phase I clinical trial (NCT03087591) has been initiated. Additionally, intravenous infusion of APN401, a suspension of Cbl-b-silenced siRNA-transfected peripheral blood mononuclear cells (PBMCs), has entered Phase II clinical trials (NCT02166255) (Gavali et al., 2021).

A pharmaceutical company named Nurix developed the most progressive program targeting CBL-B, employing a high-priority DNA-encoded library screening platform to discover compounds capable of connecting E3 ligases to specific proteins of interest. Nurix developed a Cbl-b inhibitor (NX-0255) intended for use in conjunction with adoptive T-cell therapy, known as drug-enhanced tumor-infiltrating lymphocyte (DeTIL) therapy, which is a combination therapy. This approach involves culturing tumor fragments derived from patients with IL-2 and NX-0255 to activate CD8^+^ T cells that target specific antigens. Subsequently, the modified DeTILs were reintroduced into the patient’s body. DeTIL--0255 (NCT05107739) has initiated recruitment efforts for phase I clinical trials in advanced malignancies, such as platinum-resistant ovarian cancer, endometrial cancer, and cervical cancer (Augustin et al., 2023). Another pharmaceutical company named HotSpot Therapeutics has developed a range of small molecule inhibitors targeting CBL-B, such as HOT-A, which hinders CBL-B phosphorylation and its functional activity, and is soon to upcoming clinical trials (NCT05662397) (Kuai et al., 2021).

### FBXW7

FBXW7, a member of the F-box protein family, consists of an F-box domain that serves the role of enlisting SCF complexes via Skp1, a WD40 domain that recognizes and interacts with substrates, and a D domain. FBXW7 participates in the polarization of tumor-associated macrophages through various pathways, thereby influencing tumor progression. Moreover, FBXW7 can ubiquitinate and degrade Eyes absent homolog 2 (EYA2), leading to increased immunogenicity of cancer cells, reduced carcinogenicity, and increased infiltration of natural killer (NK) cells and cytotoxic T cells (Xing et al., 2022). A related study showed that high expression of FBW7 leads to ubiquitination and degradation of the PD-1 protein, which in turn promotes the blockade of the PD-1/PD-L1 immune evasion pathway in non-small cell lung cancer (Liu et al., 2022a). *FBXW7* is one of the top 10 mutated genes commonly found in metastatic RCC cells, and it can also mediate the degradation of NFAT1, which can improve PD-L1 expression by increasing TNF levels in RCC, fostering RCC proliferation, and modulating immune responses through diverse signaling pathways (Liu et al., 2022b). Thus, drugs targeting FBXW7 have significant potential for treating renal cancer.

To date, no drugs that directly target FBXW7 have been developed. miR-223 is overexpressed in cancer cells and can downregulate the expression of FBXW7, contributing to the development of resistance to multiple anticancer drugs (Wang et al., 2023). A related study showed that genistein influences the biological characteristics of pancreatic cancer cells by elevating FBXW7 expression through the inhibition of miR-223 (Ma et al., 2013). Furthermore, treatment with decitabine, *in vitro* lung cancer cells, and *in vivo* lung cancer xenograft mouse models reverted the FBXW7 promoter to a non-methylated state, resulting in increased expression of FBXW7 (DiNardo et al., 2018). Moreover, Polo-like kinase 1 (PLK1) stimulates the phosphorylation and degradation of FBXW7,and a related study found that under the combination of Bcl-2 (ABT199) and PLK1 (BI2536) inhibitors, tumor cells exhibiting low expression of FBXW7 and overexpression of c-MYC can induce RCC cells into apoptosis (Fan et al., 2022). These findings reveal the potential of targeting FBXW7 in cancer therapy, emphasizing the need for further development of FBXW7 inhibitors and their application in RCC treatment.

## The role of deubiquitylase in RCC therapy

### USP7

USP7, a member of the ubiquitin-specific processing protease family, consists of a catalytic core domain, five C-terminal ubiquitin-like domains (UBL1-5) and an N-terminal Tumor necrosis factor Receptor-Associated Factor (TRAF)-like domain. The TRAF-like domain recognizes ubiquitylated substrates under the influence of UBL domains 4 and 5, facilitating their interaction (Gao et al., 2023).

Multiple studies have indicated that the regulatory effect of USP7 on PD-L1 expression is complex and may depend on the tumor microenvironment. In gastric cancer, USP7 expression was positively correlated with PD-L1 expression. Hence, this might be attributed to USP7’s ability to deubiquitinate and stabilize PD-L1, consequently playing a role in tumor immune evasion (Gao et al., 2023). However, a related study showed that inhibiting USP7 increased the expression of PD-L1 in lung cancer cells (Dai et al., 2020), and higher levels of USP7 promoted tumor growth by altering the immunosuppressive characteristics of Forkhead box protein P3 (Foxp3)^+^ Treg (Gao et al., 2023). Although there has been little research on USP7 and RCC, further development and clinical trials of USP7 inhibitors in combination with PD-L1/PD-1 checkpoint inhibitors have significant potential to offer new valuable avenues for immunotherapy in RCC.

A related study showed that USP7 is a target for tumor survival and is overexpressed in numerous cancers (Chauhan et al., 2012), leading to extensive research and development efforts focused on drugs targeting USP. In a mouse CT26 xenograft model, the growth of colorectal cancer tumors significantly slowed down with the combined use of P5091 and adenovirus-based vaccines compared to the use of either drug alone. And after P5091 therapy, the level of Foxp3 in CD4^+^ T cells is downregulated, leading to the inhibition of Tregs (regulatory T cells) (Fu et al., 2019). Almac4, another crucial USP7 inhibitor, has been shown to reduce tumor cell membrane PD-L1 levels, weaken the interaction between PD-L1 and PD-1, thereby rendering GC cells more sensitive to T cell-mediated cytotoxicity (Wang Z. et al., 2021). Currently, there is relatively limited research on clinical drug trials of USP7 inhibitors. However, non-clinical trials have indicated the tremendous potential of USP7 drugs.

### CSN5

COP9 signalosome 5 (CSN5) is the fifth component of the CSN complex and consists of a Jab1/Mpr1p and Pad1p N-terminus (MPN) domain metalloenzyme (JAMM) motif, which participates in the regulation of DNA repair, influences signal transduction, and governing cell proliferation (Hannss and Dubiel, 2011). Moreover, CSN5 exhibited deubiquitinating activity. Mediated by tumor necrosis factor-alpha (TNF-α), CSN5 can deubiquitinate PD-L1 in cancer cells, maintain its stability, and escape immune surveillance (Lim et al., 2016). In addition, CSN5 can deubiquitinate Snail and heat shock protein 70 (HSP70), thereby enhancing tumor invasion and metastasis (Hu et al., 2021). Additionally, a related study showed that CSN5 is upregulated in RCC tissues and stabilizes ZEB1 expression by reducing ZEB1 ubiquitination, leading to metastasis and activation of Epithelial-to-Mesenchymal Transition in RCC cells (Zhang et al., 2017). Therefore, targeting CSN5 in cancer immunotherapy may be a potential therapeutic strategy.

Currently, there are no specific clinical applications of CSN5-targeting drugs. However, related studies have suggested that certain drugs exhibit inhibitory effects on CSN5. Curcumin can inhibit the activity of CSN5 in various types of cancer and suppress TNF-α-induced PD-L1 stability in cancer cells (Uhle et al., 2003). A related study showed that in curcumin-treated mice, the population of tumor-infiltrated activated CD8^+^ T cells increased, whereas PD-L1 decreased, contributing to enhanced anti-tumor immune responses and increased survival rates in mice. Moreover, the suppression of PD-L1 by curcumin amplified the effectiveness of the anti-CTLA4 blockade (Lim et al., 2016). Additionally, studies have demonstrated that berberine acts as an immunotherapeutic agent by enhancing anti-tumor T-cell immunity, reduces the expression of PD-L1 and promotes anti-tumor immunity in Non-Small Cell Lung Cancer by specifically binding to Glu- 76 of CSN5 and inhibiting CSN5-mediated deubiquitination activity (Liu Y. et al., 2020). Moreover, CSN5i-3, a novel selective and orally available CSN5 inhibitor, significantly inhibits SRC family signaling (such as SRC and YES), showing potential therapeutic effects in prostate cancer (Mazzu et al., 2022).

The emergence of ubiquitination modification inhibitors offers a potential new direction for improving treatment outcomes in RCC patients, especially when the application of ICIs is limited. However, it is undeniable that there is still a long way to go before ubiquitination modification inhibitors can be widely applied in clinical practice. Moreover, their limitations, such as the potential for resistance, remain largely unknown.

## The role of PROTAC in RCC therapy

PROTAC, a heterobifunctional compound, consists of an E3 ubiquitin ligase ligand, a warhead for degrading the protein of interest (POI), and a linker. The binding of PROTAC to both POI and E3 ligase helps form a ternary molecular complex because of its unique molecular structure. Subsequently, as POI comes into proximity with E3 ligase, it leads to ubiquitination of POI by E3 ligase, followed by degradation (Moon et al., 2023).

PROTAC is currently a hot topic in cancer therapy and offers numerous advantages over traditional immune therapy inhibitors. First, the scope of the application of PROTAC is broader. PROTACs can bind to any region of the POI, whereas classical inhibitors require specific binding to the target protein binding pocket. Therefore, PROTACs can be used for the degradation of undruggable proteins (Liu et al., 2022d). Secondly, PROTACs may overcome drug resistance. One related study found that tumor cells often develop resistance due to prolonged exposure to drugs, and another related study found that drug-resistant tumor cells are sensitive to PROTAC compounds, suggesting that PROTACs have the capability to eliminate drug resistance (Wang et al., 2022). Lastly, compared to the side effects of traditional inhibitors of inducing alterations in genes, PROTAC has a minor impact because PROTACs directly target the proteins, resulting in negligible effects on genes (Chen et al., 2022). Therefore, PROTACs have a significant potential for use in the treatment of RCC. Therefore, we have summarized some successful experiments involving PROTAC (Table 2).

**TABLE 2:** Successful experiments involving PROTAC and DUBTAC.

Kind	Targets	Compound	Mechanism of actio	References
PROTAC	PD-L1	PROTAC P22	translates to reducing PD-L1 protein levels in a lysosome-dependent manner	Cheng et al. (2020)
		Cyclic peptide PROTAC	targeting palmitoyltransferase	Shi et al. (2023)
	AR	SARM-nutlin PROTAC	Using nutlin-3a as the MDM2 ligand and SARM as the AR ligand	Vicente and Salvador (2022)
	MDM2	MS3227	Recruit VHL and activate p53	Alaseem (2023)
DUBTAC	F508-CFTR	NJH-2-057	stabilized by deubiquitination in an OTUB1-dependent manner	Henning et al. (2022)
	WEE1	LEB-03-144 and LEB-03-146	stabilized by deubiquitination in an OTUB1-dependent manner	Henning et al. (2022)
	F508-CFTRa nd AMPKβ2	AMPK DUBTAC	tabilized by deubiquitination in an USP7-dependent manner	Liu et al. (2024)

To date, studies on PROTACs for PD-L1 degradation are relatively advanced. One study showed that the first PROTAC designed to degrade PD-L1 uses a resorcinol diphenyl ether-based ligand for PD-L1 and CRBN E3 ligase recruiters. The study also reported various PD-L1 ligands and successfully identified PROTAC P22, with an IC50 (half-maximal inhibition concentration) of 39.2 nM, as the most effective compound for degrading PD-L1. P22 moderately reduces PD-L1 protein levels in a lysosome-dependent manner, which may contribute to its immunological effects (Cheng et al., 2020). Additionally, a recent study showed that a cyclic peptide-based PROTAC targeting palmitoyltransferase DHHC3 dramatically downregulated the expression of PD-L1 in human cervical cancer cells. This targeted degradation effect is directly proportional to the dosage and duration of treatment, with a DC50 (Median Dose Concentration) value lower than that of linear peptides (Shi et al., 2023).

MDM2 acts as an oncogenic factor in RCC, and targeting MDM2 is a promising approach for treating RCC. However, MDM2 inhibitors face challenges, such as resistance, toxic side effects, and limited efficacy. MDM2 inhibitors enhance the transcription of MDM2 mRNA, resulting in elevated MDM2 protein levels. The removal of these inhibitors can lead to an increase in MDM2 protein levels, causing rapid degradation of p53, thus limiting their therapeutic effectiveness (Alaseem, 2023). Thus, the MDM2-targeting PROTAC therapeutic approach may have better prospects for cancer treatment.

The first PROTAC to degrade MDM2 is called SARM-nutlin PROTAC, which contains nutlin-3a as the MDM2 ligand and a non-steroidal selective androgen receptor modulator (SARM) as the AR ligand and successfully degrades the androgen receptor (AR) (Vicente and Salvador, 2022). Moreover, a previous study showed that the MDM2-targeted PROTAC degrader may potentially outperform the current MDM2 inhibitors in treating triple-negative breast cancer (TNBC), a type of cancer characterized by inactivation of the p53 tumor suppressor protein. The PROTAC they designed was used to treat TNBC xenograft-bearing mice, showing targeted efficacy against tumors, with no toxicity to normal cells, significantly extending survival (Adams et al., 2023). Furthermore, MS3227, a PROTAC that recruits VHL to target MDM2, activates the p53 pathway, induces downstream target transcription, and promotes cellular apoptosis in *TP53*WT leukemia cell lines. In the treatment of Acute Myeloid Leukemia, combination therapy with MS3227 and Venetoclax can enhance efficacy (Alaseem, 2023). However, there is relatively limited research on PROTACs of MDM2 in RCC.

Speckle-type POZ protein (SPOP), consisting of an N-terminal MATH domain, internal BTB and BACK domains, and a C-terminal nuclear localization signal, functions as a substrate-binding adaptor within the CULLIN3/RING-box1 E3 ubiquitin ligase complex. SPOP can mediate substrate degradation and polyubiquitination through various pathways. Once mutated, SPOP impairs substrate binding and polyubiquitination, thereby affecting various cancer-related pathways including AR signaling, DNA repair and methylation, cancer metabolism, and immunity. The oncogenic role of SPOP in RCC has been reported, primarily resulting in abnormal accumulation in the cytoplasm of RCC cells, rather than mutations (Zhang et al., 2023). A recent study showed that the SPOP inhibitor compound 6b inhibits RCC by disrupting Liquid-Liquid Phase Separation (Dong et al., 2020). Moreover, enzalutamide, an anti-androgen, enhances SPOP-driven degradation of AR and improves the anticancer effect of the receptor tyrosine kinase inhibitor sunitinib in AR-positive RCC (Adelaiye-Ogala et al., 2018). Therefore, the treatment of RCC can be targeted through the related actions of SPOP.

Recent studies have indicated that SPOP substrates contain one or multiple SBC motifs that are involved in numerous protein-protein interactions and signaling pathways. Hence, SPOP can be used to design PROTACs for treating SPOP-overexpressing RCC (Zhuang et al., 2009). A PROTAC targeting the AR protein based on the VHL ligand has been developed to address the insufficient degradation of the AR protein in SPOP-overexpressing RCC (Liu J. et al., 2020). Considering that the majority of RCC belong to the *VHL*-defective type, the application scope of VHL-based PROTAC is quite limited. Moreover, cancers with SPOP mutations retain wild-type p53, and utilizing MDM2-based PROTAC to degrade nuclear SPOP substrates aims to target cancers characterized by SPOP mutations (Zhang et al., 2023).

## The role of DUBTAC in RCC therapy

Unlike PROTACs, which promote ubiquitination and degradation of target proteins, DUBTACs stabilize targeted proteins by deubiquitination. The structure of DUBTACs is similar to that of PROTACs, consisting of three functional parts: a ligand for the POI, a DUB ligand, and a chemical linker. DUBTACs can trigger deubiquitination of the POI by bringing the DUB into proximity with the POI, thereby enhancing the protein levels of the POI. Abnormal protein degradation can also lead to cancer, as seen with p53 in cancer cells (Chen et al., 2024). Therefore, DUBTACs have potential for stabilizing tumor suppressor proteins within cells, thereby offering a therapeutic approach for cancer treatment.

DUBTACs are currently in the early stages of development. The first DUBTAC, named NJH-2-057, consists of the OTUB1 ligand EN523 and the lumacaftor which is a small molecule drug used for the treatment of cystic fibrosis, and the NJH-2-057 significantly increases ΔF508-CFTR protein levels by reducing its ubiquitination (Henning et al., 2022). Additionally, two DUBTACs, LEB-03-144 and LEB-03-146, composed of the WEE1 inhibitor AZD1775 and the OTUB1 recruiter EN523, successfully stabilized the tumor suppressor kinase WEE1 by deubiquitination. This effect was similar to that observed when using the proteasome inhibitor bortezomib in the HEP3B liver cancer cell line (Henning et al., 2022).

**FIGURE 9:**
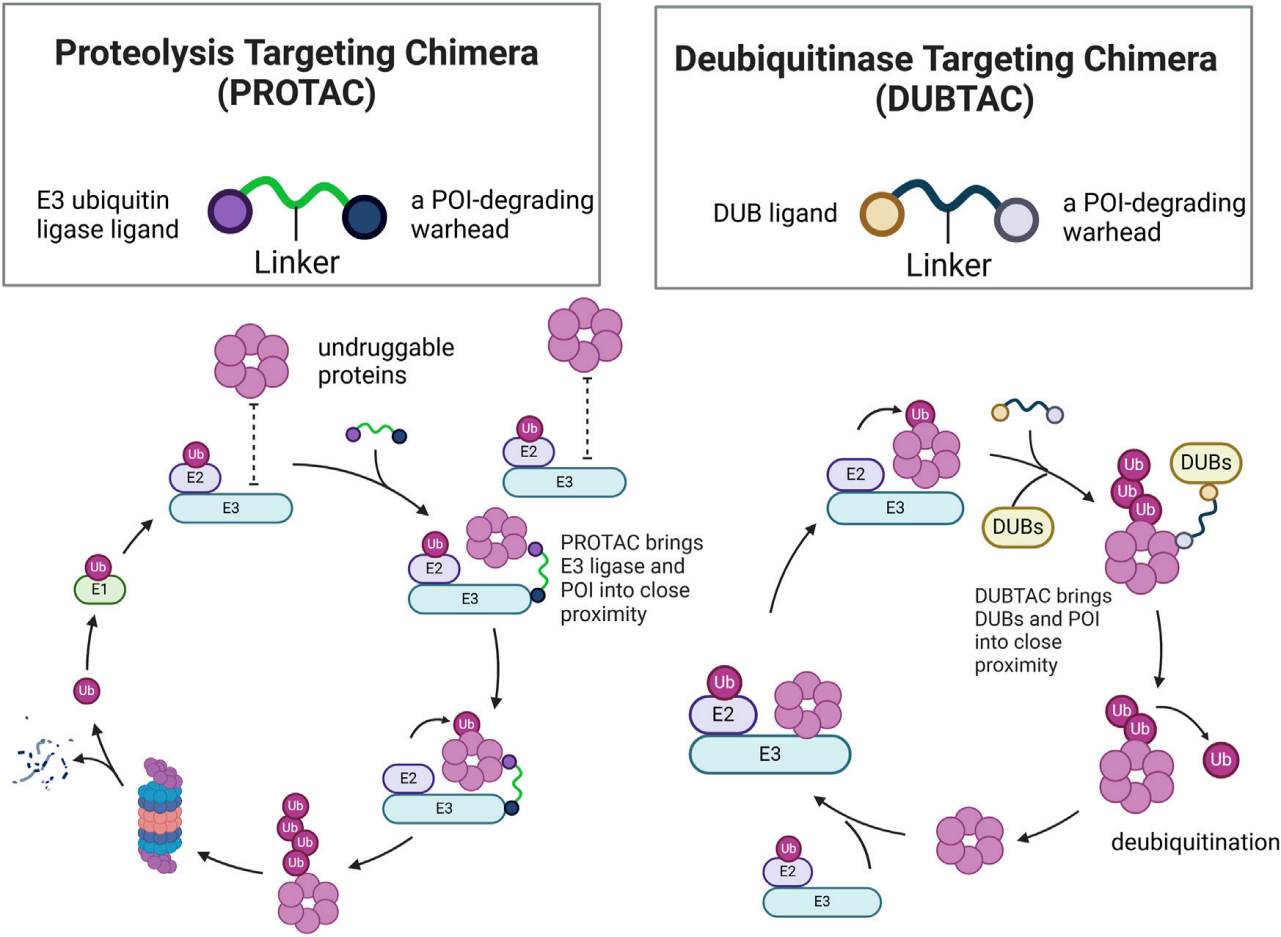
Structural comparison of PROTACs and DUBTACs and mechanistic comparison of PROTAC-Mediated ubiquitination and DUBTAC-Mediated deubiquitination of POI.

A recent study reported that the development of an AMPK DUBTAC based on a non-covalent ligand for USP7, which effectively stabilized the ΔF508-CFTR mutant protein as efficiently as the previously reported NJH-2-057. Additionally, it effectively stabilized AMPKβ2, activated AMPK signaling, and reduced tumor cell growth (Liu et al., 2024). Targeted protein stabilization is an increasingly promising approach in cancer drug development, and DUBTACs have the potential to treat RCC similarly to traditional drug inhibitors (Figure 9).

## Conclusions and perspectives

RCC is the most common form of kidney cancer, accounting for 80% of primary renal tumors, and represents the highest fatality rate among genitourinary system cancers. Conventional surgical and chemotherapy treatments have shown significant efficacy in patients in early localized stages. However, for those with distant metastasis, targeted therapy is crucial for the management of RCC patients (Weaver et al., 2022). Due to the crucial impact of ubiquitination on various cellular processes, such as proliferation, apoptosis, and DNA repair, the relationship between dysregulation of ubiquitination and RCC tumorigenesis and drug resistance has gained increasing attention. This review introduces the roles of certain ubiquitin ligases, including VHL, HAF, β-TrCP, and MDM2, as well as deubiquitinases such as BAP1,USP37 and so on. Interestingly, the same ubiquitin ligase regulates multiple signaling pathways, such as VHL involvement in the HIF signaling pathway, ferroptosis signaling pathway, PI3/AKT signaling pathway, and p53 signaling pathways. Moreover, we provide a comprehensive summary of RCC-associated ubiquitination modification in tumorigenesis and drug resistance, as well as the potential targets introduced.

We will also introduce some additional targeted ubiquitination modification inhibitor cases, although currently not studied for treating RCC, and offer new possibilities for targeted USP therapy to treat cancer. Lee et al. discovered that IU1, a USP14 inhibitor, inhibited USP14 activity and significantly reduced the viability and migration capabilities of gastric cancer cells (Lee et al., 2023). Chen et al. found that WP 1130, an inhibitor of USP9X, inhibited cell proliferation and tumor sphere formation in glioblastoma cells and successfully prolonged the survival of tumor-bearing mice treated with WP 1130 (Kong and Jin, 2024). CC-90009, an E3 complex CRL4CRBN regulator, targets GSP1 for ubiquitination and proteasomal degradation, leading to rapid depletion of GSP1 and induction of apoptosis in AML cells, thereby reducing leukemia engraftment and leukemia stem cell numbers in primary AML patients (Xiao et al., 2022). In addition, several inhibitors of ubiquitination modification are currently undergoing clinical studies. SAR-405838, CGM097, and DS3032b, which target MDM2, are presently in Phase I clinical trials for patients with advanced solid tumors. Novantrone, a compound that inhibits USP11, is being tested in Phase I-IV clinical trials for T-cell lymphoma, AML, multiple sclerosis, and breast cancer (Park et al., 2020).

A large of studies have found that ubiquitination modification plays a crucial role in the progression of RCC. Ubiquitin ligases and deubiquitinases regulate key proteins in multiple signaling pathways. Once ubiquitination is dysregulated, normal renal cells may transform into RCC, or RCC cells may undergo rapid progression. Given that monotherapy and traditional small-molecule kinase inhibitors are easy to resist, and targeting ubiquitin ligases presents a promising strategy for RCC treatment. Research on drugs targeting ubiquitin ligases or ubiquitinases in RCC is underway, but most drugs are still in preclinical stages. Transitioning these findings into clinical applications remains a challenge. Perhaps identifying new ubiquitination modification targets in RCC and developing more suitable inhibitory drugs could also be a promising direction. Given the success of PROTAC in treating other cancers, developing novel PROTACs targeting ubiquitin ligases or deubiquitinases involved in RCC regulation and applying them to RCC therapy represents a promising direction for future research.
